# Aromatase, testosterone, TMPRSS2: determinants of COVID-19 severity

**DOI:** 10.1186/s13293-024-00658-4

**Published:** 2024-10-24

**Authors:** Eric C. Mohan, Jude P. J. Savarraj, Gabriela D. Colpo, Diego Morales, Carson E. Finger, Alexis McAlister, Hilda Ahnstedt, HuiMahn Choi, Louise D. McCullough, Bharti Manwani

**Affiliations:** 1 Department of Neurology, University of Texas Health Science Center at Houston, McGovern Medical School, Houston, TX 77030 USA; 2 Vivian L. Smith Department of Neurosurgery, University of Texas Health Science Center at Houston, McGovern Medical School, Houston, TX 77030 USA

**Keywords:** COVID-19, Sex differences, CRP, Aromatase

## Abstract

**Background:**

Male sex has been identified as a risk factor for worse COVID-19 outcomes. This sex difference has been mostly attributed to the complex role of sex hormones. Cell surface entry of SARS-CoV-2 is mediated by the transmembrane protease serine 2 (TMPRSS2) which is under transcriptional regulation by androgens. P450 aromatase enzyme converts androgens to estrogens. This study measured concentrations of aromatase enzyme, testosterone, estradiol, and TMPRSS-2 in plasma of hospitalized COVID-19 patients to elucidate the dynamics of sex-linked disparity in COVID-19 and correlate them with disease severity and mortality.

**Methods:**

In this prospective cohort study, a total of 265 patients (41% women), age 18 years and older, who had a positive COVID-19 PCR test and were hospitalized for COVID-19 at Memorial Hermann Hospital in Houston, (between May 2020 and May 2021) were enrolled in the study if met inclusion criteria. Plasma concentrations of Testosterone, aromatase, TMPRSS-2, and estradiol were measured by ELISA. COVID-19 patients were dichotomized based on disease severity into moderate-severe (*n* = 146) or critical (*n* = 119). Mann Whitney U and logistic regression were used to correlate the analytes with disease severity and mortality.

**Results:**

TMPRSS2 (2.5 ± 0.31 vs. 1.73 ± 0.21 ng/mL, *p* < 0.01) and testosterone (1.2 ± 0.1 vs. 0.44 ± 0.12 ng/mL, *p* < 0.01) were significantly higher in men as compared to women with COVID-19 after adjusting for age in a multivariate model. There was no sex difference seen in the level of estradiol and aromatase in COVID-19 patients. TMPRSS2 and aromatase were higher, while testosterone was lower in patients with increased COVID-19 severity. They were independently associated with COVID-19 severity, after adjusting for several baseline risk factors in a multivariate logistic regression model. In terms of mortality, TMPRRS2 and aromatase levels were significantly higher in non-survivors.

**Conclusions:**

Our study demonstrates that testosterone, aromatase, and TMPRSS2 are markers of COVID-19 severity. Estradiol levels do not change with disease severity in COVID-19. In terms of mortality prediction, higher aromatase and TMPRSS-2 levels can be used to predict mortality from COVID-19 in hospitalized patients.

**Plain English Summary:**

COVID-19 has caused over a million deaths in the U.S., with men often getting sicker than women. Testosterone, a male hormone, helps control a protein called TMPRSS-2, which allows the COVID-19 virus to spread more easily in the body. A protein called aromatase converts the male hormone testosterone into the female hormone estrogen. It is thought that female hormone estrogen helps protect women from getting seriously ill from COVID-19. To understand the role of these hormones in COVID-19 and sex differences, we measured levels of testosterone, estrogen, aromatase (which turns testosterone into estrogen), and TMPRSS-2 in hospitalized COVID-19 patients. We also checked how this level might reflect the severity of the disease. We found that critically ill COVID-19 patients (the ones in ICU) had higher levels of TMPRSS-2 and aromatase, and lower testosterone levels. When we used these hormone levels to predict death in hospitalized COVID-19 patients, higher levels of TMPRSS-2 and aromatase were linked to a lower chance of survival.

## Highlights

COVID-19 Disease Severity: In hospitalized patients with COVID-19, higher TMPRSS-2, aromatase and lower total testosterone are markers of disease severity.

COVID-19 Mortality: In hospitalized patients with COVID-19, TMPRSS2 and aromatase levels are significantly increased in COVID-19 non survivors.

## Introduction

Coronavirus disease 19 (COVID-19), caused by severe acute respiratory syndrome coronavirus 2 (SARS-CoV-2) has led to over 1 million deaths in the United States [1]. Initial studies identified age as a risk factor for symptomatic infection, as seen with other respiratory illnesses [2]. Meta-analyses of 70 clinical reports from around the world, detailing COVID-19 infections by sex, have shown that while the number of infected men (47.9%) and women (51.5%) were similar, men had a higher mortality rate, 56.1% as compared to women, 42.5% [3]. In intensive care unit admissions, women had significantly fewer admissions, 30.4% compared to men, 69.6% [3]. Therefore, male sex has been recognized to be a risk factor for COVID-19 severity [4]. This may be due to the complex role of sex chromosomal complement or epigenetic factors, but a link with sex hormones has appeared to be more plausible [5]. This is related to the mechanism of entry for the SARS-CoV-2 virus. Cell surface entry of SARS-CoV-2 is mediated by the transmembrane protease serine 2 (TMPRSS2). The spike protein of the SARS-CoV-2 virus binds to TMPRSS2 on the cell surface where it is then cleaved by TMPRSS2, leading to membrane fusion of the viral capsid with the eukaryotic membrane, internalizing the free-floating viral RNA into the cytoplasm [6]. Interestingly, hormone testosterone has been shown to regulate TMPRSS2 expression in different human and animal models [7]. Also, in vitro studies of A549 human lung cancer cells have shown that 24 h of testosterone treatment results in the upregulation of the TMPRSS2 gene, upregulating TMPRSS2 protein on the cell surface [8]. Interestingly, testosterone concentration is regulated by the enzyme P450 aromatase (aromatase) that converts testosterone to 17β-estradiol via a sequence of redox, dehydration, and elimination reactions [9]. Aromatase’s role is critical to sexual differentiation [10]. In peripheral blood, aromatase is synthesized by blood leukocytes and helps modulate sex hormone concentrations in circulation [8]. On the other hand, the female sex hormone, estrogen has been known to have anti-inflammatory and protective effects in COVID-19 [11]. Many studies have focused on quantifying the level of these hormones individually in small cohorts of only men or women. To understand the dynamics of aromatization and the role of sex hormones, the goal of this study was to quantify the endogenous sex hormones (estradiol, testosterone, and aromatase) in hospitalized COVID-19 patients. Another secondary objective was to determine the association of the levels of these analytes with COVID-19 outcomes (disease severity, in-hospital mortality).

## Methods

### Study population and patient inclusion/exclusion criteria

This study screened 3045 patients who were admitted to Memorial Hermann Hospital, Houston, Texas, USA with a positive nasopharyngeal swab confirmed by real-time polymerase chain reaction for COVID-19 between May 2020 to May 2021 [12, 13]. Of the 3045 patients, we excluded 2377 patients in which COVID-19 was not the primary diagnosis for admission but was incidental. Patients with steroids, hormone replacement therapy, immunosuppressants, or chemotherapy on home medication history (*n* = 431) were further excluded from the study. 265 patients met our inclusion and exclusion criteria, and peripheral blood samples were collected after patient consent. 33% of the patients were admitted to the ICU. Patients were then categorized based on WHO COVID-19 severity classification, as either moderate-severe (hospitalized and requiring supplemental oxygen) or critical (in ICU on ventilator/ artificial life support) [2]. All procedures were approved by the Institutional Review Board at The University of Texas Health Science Center at Houston, Houston, Texas, USA.

### Sample collection

Patients were consented and enrolled within 48 h of admission. Blood samples were collected fasting, with morning labs, around 4–5 am for all patients. Peripheral blood was collected in appropriate sterile vacutainers and transported on ice for processing. Blood plasma was isolated by centrifuging samples at 1,200 x g for 10 min at 4 °C, followed by plasma supernatant isolation and further centrifugation at 10,000 x g for 10 min at 4 °C to generate plasma. Samples were stored at -80 ˚C in aliquots until analysis.

### ELISA analysis

We used enzyme-linked immunosorbent assay (ELISA) to measure the levels of total testosterone (Millipore Sigma, Inc, Burlington, MA, USA), aromatase (Cloud-Clone Corp, Houston, TX, USA), TMPRSS2 (Novus, Centennial, CO, USA), and estradiol (Calbiotech, El Cajon, CA, USA). CRP values were obtained from hospital records at the time of admission.

### Statistical analysis

Descriptive statistics were calculated for demographic variables and hormone levels in control and COVID-19 subjects. Results are reported as mean ± standard error of the mean (SEM). To describe differences in demographics, χ2-test, Fisher’s exact test, student’s t-test, and the Mann-Whitney U test were used where appropriate. The Mann-Whitney U test was used to test for differences in analyte levels across different groups. A p-value of ≤ 0.05 was considered statistically significant. In constructing the multivariate models, we selected variables based on their relevance to COVID-19 severity and mortality, as well as their statistical significance within our dataset [14]. Logistic regression (LR) method was used, and the predictive performance was measured using area under the curve (AUC) analysis. The receiver operating characteristics (ROC) curves were computed and the area under the AUC of each model was obtained. The ROC curves were compared using the De-Long method [15]. Figures were made using GraphPad Prism version 8. All statistical analyses were performed using open-source software packages in R (v3.1.3) and MedCalc for Windows, version 15.0 (MedCalc Software, Ostend, Belgium).

## Results

### Sex Differences in COVID-19

We analyzed samples from 265 hospitalized COVID-19 patients, where 41% of the COVID-19 patients were women. The mean age of women was 54.3 years, (range, 19 to 95 years), with 58.7% of women enrolled being above 51 years (average age of menopause [16]). The mean age of men was 52.8 years of age, (range, 22 to 101 years), with 56.4% of men enrolled being over the age of 51 years. There was no significant difference in the demographics, past medical history, and mortality between men and women with COVID-19 in the hospitalized patients (Table 1: Demographics of Hospitalized COVID-19 Patients; Women vs. Men). CRP levels after COVID-19 admission were significantly higher in men compared to women (107 ± 7 vs. 86 ± 7.3 mg/L, *p* < 0.05). Plasma samples were available from 109 women and 156 men, and the availability of sex hormone levels are as follows: testosterone for 182 samples (53 women and 129 men): TMPRSS2 for 220 samples (92 women and 128 men), estradiol for 108 samples (44 women and 64 men) and aromatase for 259 samples (108 women and 151 men).

**Table 1:** Demographics of Hospitalized COVID-19 Patients; Women vs Men. Sex differences in testosterone, TMPRSS2, Estradiol and aromatase levels were assessed. Testosterone and TMPRSS2 were significantly higher in men vs. women

	Women (n = 109)	Men (n = 156)	p-value	Sample Size
**Demographics**				
Age (mean,sd)	54.3(16)	52.8(15.7)	0.4	
Race (n,%)				
African American	22(20)	26(16.6)	0.35	
White	53(48)	84(53)	0.47	
Other	34(31)	46(29)	0.87	
Ethnicity, Hispanic (n,%)	62(56)	93(59)	0.75	
**Past Medical History**				
Hypertension (n,%)	51(46)	74(47)	1	
Diabetes (n,%)	41(37)	56(36)	0.87	
Hyperlipedimia (n,%)	20(18)	32(20)	0.78	
Obesity (n,%)	59(54)	82(52)	0.89	
Smoking(n,%)	5(4.5)	18(11)	0.07	
Mortality at discharge (n,%)	25(23)	37(23)	0.99	
**Hormones**				
Testosterone, ng/ml (mean ± sem)	0.44 ± 0.12	1.2 ± 0.1	< 0.01*	n = 182 (153 vs 129)
TMPRSS2, ng/ml (mean ± sem)	1.73 ± 0.21	2.5 ± 0.31	0.04*	n = 220 (92 vs 128)
Estradiol, pg/ml (mean ± sem)	48 ± 8.3	53 ± 7.75	0.66	n = 108 (44 vs 64)
Aromatase, ng/ml (mean ± sem)	7 ± 0.42	7.3 ± 0.27	0.4	n = 259 (108 vs 151)

* Significant after adjusting for age

TMPRSS2 (2.5 ± 0.31 vs. 1.73 ± 0.21 ng/mL, *p* < 0.01) and testosterone (1.2 ± 0.1 vs. 0.44 ± 0.12 ng/mL, *p* < 0.01) were both significantly higher in men compared to women. Both were independently associated with sex after adjusting for age in a multivariate model (Table 1). There was no sex difference seen in the level of estradiol and aromatase in the hospitalized COVID-19 patients.

### Clinical Severity and Sex Hormones

COVID-19 patients were dichotomized based on disease severity into either moderate-severe (*n* = 146) or critical (*n* = 119) disease. Patients with critical disease were older and had higher CRP levels at admission (Table 2: Demographics of Hospitalized COVID-19 Patients; Moderate-Severe vs. Critical). There were no differences in comorbidities, history of hypertension, diabetes, hyperlipidemia, obesity, or smoking between the two groups. We analyzed levels of sex hormones stratified by critical vs. moderate-severe subjects. TMPRSS2 (3.46 ± 0.38 vs. 1.15 ± 0.12 ng/mL, *p* < 0.01), estradiol (67 ± 9.6 vs. 34 ± 4.8 pg/mL, *p* < 0.01) and aromatase (7.9 ± 0.4 vs. 6.5 ± 0.24 ng/mL, *p* < 0.01) were significantly higher and testosterone (0.7 ± 0.1 vs. 1.27 ± 0.13 ng/mL, *p* < 0.01) was significantly lower in patients with critical disease, as compared to the ones with moderate-severe disease. Testosterone, TMPRSS2, and aromatase were independently associated (*p* < 0.01) with severity after adjusting for several baseline risk variables (including age, sex, CRP, obesity, hypertension, hyperlipidemia, and diabetes) in a multivariate logistic regression model (Table 2).

**Table 2:** Demographics of Hospitalized COVID-19 Patients; Moderate-Severe vs. Critical. Testosterone was decreased and TMPRSS2, Aromatase were significantly increased with disease severity after adjusting for age, sex, CRP, obesity, hypertension, hyperlipidemia, diabetes mellitus (‡)

	Moderate-Severe (n = 146)	Critical (n = 119)	p-value	Sample Size
**Demographics**				
Age (mean,sd)	51(15)	56(16.8)	0.007	
Sex (female,%)	67(46)	42(35)	0.1	
**Past Medical History**				
Hypertension (n,%)	67(45)	58(48.7)	1	
Diabetes (n,%)	52(35.6)	45(38)	0.8	
Hyperlipedimia (n,%)	29(19.8)	23(19)	1	
Obesity (n,%)	76(52)	65(54)	0.89	
Smoking (n,%)	14(9.5)	9(7.5)	0.6	
**Severity**				
CRP,mg/L (mean ± se)	55 ± 3.2	152 ± 8.4	< 0.01	
Mortality at discharge (n,%)	0(0)	62(52)	< 0.01	
**Hormones**				
Testosterone, ng/ml (mean ± sem)	1.27 ± 0.13	0.7 ± 0.1	< 0.01*~‡	n = 182 (96 vs 86)
TMPRSS2, ng/ml (mean ± sem)	1.15 ± 0.12	3.46 ± 0.38	< 0.01*~‡	n = 220 (123 vs 97)
Estradiol, pg/ml (mean ± sem)	34 ± 4.8	67 ± 9.6	< 0.01*	n = 108 (152 vs 56)
Aromatase, ng/ml (mean ± sem)	6.5 ± 0.24	7.9 ± 0.4	< 0.01*~‡	n = 259 (146 vs 113)

*Significant after adjusting for Age and Sex

~Significant after adjusting for Age, Sex and CRP

‡Significant after adjusting for Age, Sex, CRP, Obesity, Hypertension, Hyperlipidemia, Diabetes Mellitus

**Fig. 1:**
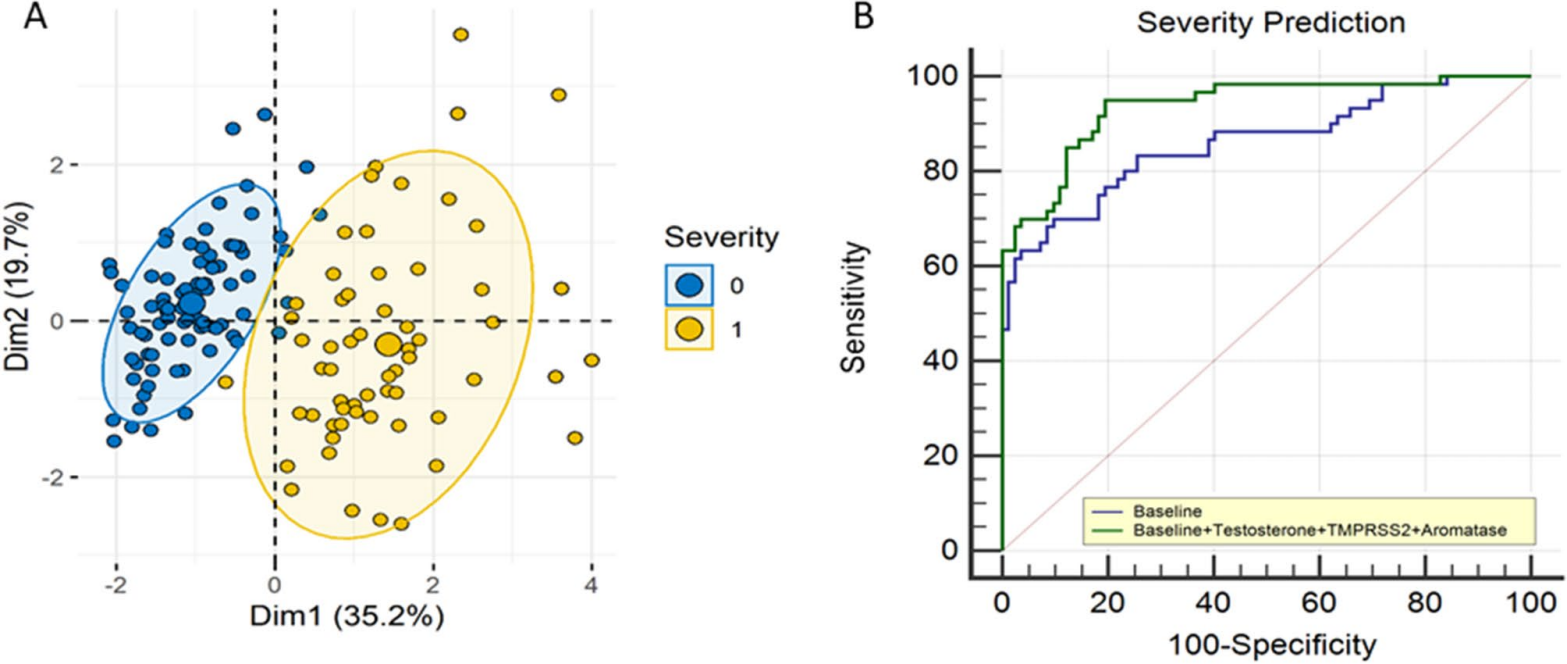
Clinical severity and Hormones: (**A**) Using PCA analysis, we report a panel of hormones (testosterone, TMPRSS2 and Aromatase) was able to segregate COVID-19 patients who ate at risk of severe clinical course (1-Clinical, 0-Moderate-Severe) (**B**) Inclusion of hormone panel (including testosterone, TMPRSS2 and Aromatase) in addition to baseline risk factors in a logistic regression model significantly imporved the prediction of severity course over the baseline model by 8.7% (0.94 ± 0.02 [95% CI: 0.88 to 0.97] vs 0.86 ± 0.03 [95% CI: 0.79 to 0.91], p<0.01, De-Long Test)

Given a significant difference in hormone levels was seen with severity, we next investigated if any of these hormones can predict COVID-19 severity (Fig. 1: **Clinical Severity and Hormones)**. PCA analysis with baseline variables (including age, sex, and CRP) and hormones (testosterone, TMPRSS2, and aromatase) indicated excellent segregation between patients with moderate-severe vs. critical disease (Fig. 1A). We developed two multivariate models: a baseline model (including age, sex, CRP, obesity, hypertension, hyperlipidemia, and diabetes) and a hormone model (that included the testosterone, TMPRRS2, and aromatase level in addition to the variables in the baseline model). The baseline model included variables that are known predictors of adverse COVID-19 outcomes, such as age, CRP levels, hypertension, hyperlipidemia, and diabetes mellitus. We performed univariate analysis on these known variables, and only those with a p-value < 0.1 were included in the multivariate model (age, hypertension, diabetes, hyperlipidemia, and CRP were all *p* < 0.05 in the univariate model).

Although several studies have identified smoking as a risk factor for adverse COVID-19 outcomes, in our cohort, smoking was not significantly associated with mortality in a univariate analysis (*p* = 1.0). The prevalence of smoking was low among both survivors and non-survivors (7% vs. 7.8%), leading to its exclusion from the multivariate model to preserve model parsimony. The hormone model significantly improved the prediction of clinical severity compared to the baseline model by 8.7% (0.94 ± 0.02 [95% CI: 0.88 to 0.97] vs. 0.86 ± 0.03 [95% CI: 0.79 to 0.91], *p* < 0.01, De-Long Test) (Fig. 1B).

### Mortality and sex hormones

In-hospital mortality data were extracted for these patients hospitalized with COVID-19. No mortality was reported in moderate-severe COVID-19 patients. Critical COVID-19 patients were dichotomized based on mortality: Non-survivors (*n* = 64) vs. survivors (*n* = 55) as per discharge disposition. Mortality was higher with advanced age, co-morbidity of hypertension, and hyperlipidemia (Table 3: Mortality Analysis of Critical COVID-19 Patients). Interestingly, among the patients with critical disease, the survivors had significantly higher CRP levels than non-survivors (182 ± 9.3 vs. 127 ± 16 mg/L, *p* < 0.1). There was no difference in diabetes, obesity, and smoking in the two groups. We analyzed the levels of hormones stratified by mortality in the patients that had critical COVID-19 and found that TMPRSS2 (4.3 ± 0.59 vs. 2.3 ± 0.4 ng/mL, *p* < 0.01), estradiol (81 ± 13.7 vs. 43 ± 10.6 pg/mL, *p* = 0.05) and aromatase (9.3 ± 0.74 vs. 6.4 ± 0.3 ng/mL, *p* < 0.01) were significantly higher in non-survivors. Testosterone was higher in survivors, but this trend was not statistically significant (*p* = 0.33). TMPRSS2 and aromatase were both increased in non-survivors and independently associated (*p* < 0.01) with mortality after adjusting for several baseline risk variables (including age, sex, CRP, hypertension, hyperlipidemia, and diabetes) in a multivariate logistic regression model.

**Table 3:** Mortality Analysis of Critical COVID-19 Patients. TMPRSS2 and aromatase were significantly increased in survivors vs. non-survivors, after adjusting for age, sex, CRP, obesity, hypertension, hyperlipidemia, diabetes mellitus (‡)

	Survivors (n = SS)	Non-Survivors (n = 64)	p-value	Sample Size
**Demographics**				
Age (mean,sd)	50.4(15.8)	61.5(16)	< 0.01	
Sex (female,%)	16(29)	26(40)	0.2	
Race (n,%)				
African American	7(12)	11(17)	0.67	
White	31(56)	32(50)	0.61	
Other	17(31)	21(32)	0.98	
Ethnicity, Hispanic (n,%)	38(69)	41(64)	0.7	
**Past Medical History**				
Hypertension (n,%)	21(38)	37(57)	0.05	
Diabetes (n,%1	16(291	29(451	0.1	
Hyperlipidemia (n,%)	5(9)	18(28)	0.01	
Obesity (n,%)	34(62)	31(48)	0.2	
Smoking (n,%)	4(7)	5(7.8)	1	
**Severity**				
CRP,mg/L (mean ± se)	182 ± 9.3	127 ± 12	< 0.01	
**Hormones**				
Testosterone, ng/ml (mean ± sem)	0.84 ± 0.15	0.64 ± 0.12	0.33	n = 86 (42 vs 44)
TMPRSS2, ng/ml (mean ± sem)	2.3 ± 0.4	4.3 ± 0.59	< 0.01*‡	n = 97 (41 vs 56)
Estradiol, pg/ml (mean ± sem)	43 ± 10.6	81 ± 13.7	0.05	n = 56 (22 vs 34)
Aromatase, ng/ml (mean ± sem)	6.4 ± 0.3	9.3 ± 0.74	< 0.01*~‡	n = 113 (54 vs 59)

*Significant after adjusting for Age

~Significant after adjusting for Age, Hypertension, Hyperlipidemia, Diabetes Mellitus

‡Significant after adjusting for Age, CR P, Hypertension, Hyperlipidemia, Diabetes Mellitus

**Fig. 2:**
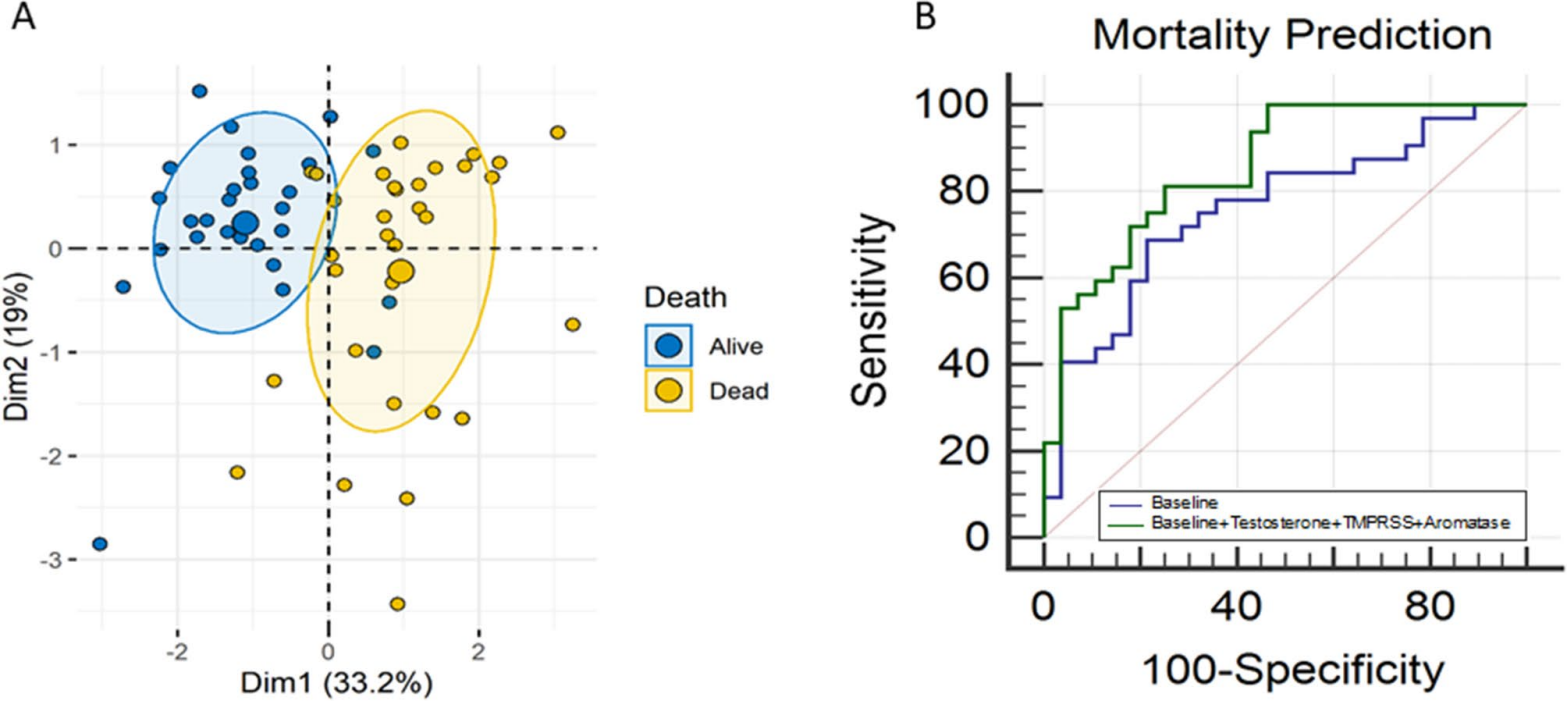
Mortality and Hormones: (**A**) Using PCA analysis, tested the same panel of hormones (inludng testosterione, TMPRSS2, and Aromatase) used to segregate COVID-19 severity to segregate mortality among critical subjects. (**B**) The same panel of hormones in addition to baseline risk factors improved the prediction of mortality at admission over the baseline model by 13% (0.86 ± 0.04 [95% CI: 0.74 to 0.93] vs 0.76 ± 0.06 [95% CI: 0.63 to 0.86], p<0.01, De-Long Test

We investigated if the hormone levels on admission can predict mortality (Fig. 2: **Mortality and Hormones**). PCA analysis with baseline variables (including age and CRP) and hormones (testosterone, TMPRSS2, and aromatase) indicated reasonable segregation between survivors and non-survivors (Fig. 2A). We developed two multivariate models: a baseline model (including age, sex, CRP, hypertension, hyperlipidemia, and diabetes) and a hormone model (that included the testosterone, TMPRRS2 and aromatase levels at admission in addition to the variables in the baseline model). The hormone model significantly improved AUC over the baseline model by 13% (0.86 ± 0.04 [95% CI: 0.74 to 0.93] vs. 0.76 ± 0.06 [95% CI: 0.63 to 0.86], *p* < 0.01, De-Long Test (Fig. 2B).

## Discussion

Our study was designed to measure the levels of sex steroids in hospitalized COVID-19 patients at admission and associate them with disease severity and mortality. We found that P450 aromatase, testosterone, and TMPRSS-2 can be markers of COVID-19 severity. Additionally, TMPRSS-2 and aromatase can be predictors of mortality in patients with critical COVID-19 disease. TMPRSS-2 is a transmembrane serine protease that facilitates SARS-COV-2 viral entry in target cells [17–19], and androgen receptors are transcription promotors for this protease [17]. Thus, androgens like testosterone activate the androgen receptor, promoting TMPRSS2 transcription and increased viral entry into cells. Higher levels of TMPRSS-2 have been postulated to be the etiology of increased disease severity in men with COVID-19 [20, 22] contributing to the observed sex differences in COVID-19. In our enrolled patients who were hospitalized with COVID-19, there were higher levels of testosterone and TMPRSS-2 in men as compared with women, consistent with prior studies [23]. Estradiol and aromatase levels did not differ between sexes in patients with COVID-19, which may be secondary to the enrollment of more post-menopausal women in our study (58.7% were above 51 years old). Patients with critical disease had higher levels of aromatase, TMPRSS2, and lower levels of testosterone as compared to patients with moderate-severe disease. This suggests that TMPRSS2, testosterone, and aromatase can be used as markers of poor prognosis or increased disease severity in COVID-19 hospitalized patients.

Cytochrome P450 aromatase is an enzyme involved in the biosynthesis of estrogens and sexual differentiation [24]. It converts androgens into estrogens maintaining the balance of sex hormones in the body and its activity has been detected in ovaries, adipose tissue, brain, breast, and placenta [25]. Our analysis of patients with COVID-19 has revealed a significant increase in aromatase levels and decreased testosterone levels in patients with critical disease, suggesting increased aromatase activity with disease severity. This is consistent with a recent study that used exome sequencing and demonstrated that CYP19A1 (aromatase) activity increasing mutation Thr201Met is associated with increased disease severity in men [26]. This is the first study to measure protein aromatase in hospitalized COVID-19 patients (both men and women) and demonstrate its association with disease severity. It is speculated that increased aromatization of androgens to estrogens may be the protective response to SARS-COV-2. We found a trend, but no significant increase, in estradiol levels in patients with severe COVID-19 after controlling for variables such as age, sex, and comorbidities. Estradiol, a sex steroid is known to have anti-inflammatory effects in COVID-19 [27]. Sex differences in COVID-19 outcomes have been attributed to these protective effects of estrogens. In a Sweden-based registry, estrogen supplementation in post-menopausal women was associated with decreased mortality from COVID-19 [28, 29]. This can be due to the anti-inflammatory effects of estrogens, for example, reduction in proinflammatory cytokines, increased T cell response, and increased antibody production from B Cells [28]. Aromatization of androgens to estrogens is a dynamic process, and it is possible that the two-day post-admission time point was too early to detect significant changes in estradiol levels in our study. It is also possible that the elevation of estradiol may be at the tissue level and the measured plasma estradiol may not be reflective of the tissue level increase. Future preclinical studies can provide more insights into the dynamics of this sex-hormonal milieu.

Consistent with several other studies [30–33], testosterone concentrations in patients with critical COVID-19 were found to be significantly lower than those in moderate-severe disease patients. This further supports the theory that, in severe inflammatory states caused by COVID-19, testosterone is actively converted to estradiol by aromatase as a compensatory defense mechanism. Alternatively, the reduction in testosterone levels may represent transient hypogonadism or due to transient fasting, a stress response that has also been documented in other severe illnesses [34, 35]. TMPRSS2 levels increased with disease severity in patients with COVID-19, consistent with prior reports [7, 19, 36, 37]. Testosterone can upregulate TMPRSS2, a protease that is important for viral entry and activation [38]. The paradoxical decrease in testosterone levels with increased TMPRSS2 levels in patients with severe COVID-19 can potentially be secondary to negative feedback inhibition. This further highlights the complex dynamics of sex steroid aromatization and androgen receptor activity on TMPRSS2. Longitudinal studies at different time points may help further understand the role of this aromatization pathway.

## Limitations

Our study had some limitations. Being a single-center study, it was designed to predict associations of sex steroids, aromatase, and TMPRSS2 levels with disease severity and mortality. Association does not imply causation; therefore, these results should be interpreted with caution. Due to the limited availability of human plasma for each analyte, the number of patients tested for each analyte varied. Moreover, when the data was dichotomized based on disease severity, our study lacked a sufficient sample size to perform a sex-based subgroup analysis. All our enrolled patients were hospitalized, so results should be interpreted carefully as they pertain to moderate-severe and critical cases of hospitalized patients and not to COVID-19 patients that recover in the outpatient and do not need supplemental oxygen (classified as mild per WHO classification). Furthermore, 58.7% of the women in our study were above 51 years, potentially peri or post-menopausal, which may affect the levels of estradiol. Although the analysis of analytes in our hospitalized patients may not change with vaccination or COVID-19 strain, it is important to realize that our study participants were enrolled between May 2020 and May 2021 when most patients were unvaccinated and the dominant strains were the original strain, D614G and B.1.17 mutant. We measured these markers at a single time point, within 48 h of admission, using ELISA. Future studies incorporating measurements at multiple time points and utilizing more advanced techniques like mass spectrometry may provide deeper insights into the complex dynamics of these hormones and markers.

## Conclusion

This study shows that testosterone, aromatase, and TMPRSS2 are markers of COVID-19 severity in hospitalized patients. Estradiol levels do not change with disease severity in COVID-19. In terms of mortality prediction, high aromatase and TMPRSS-2 levels measured within 48 h of hospital admission can be used to predict mortality from COVID-19.

## Data Availability

Data available from the corresponding author upon request.

## References

[1] Le T-T, Liao X. Two-part predictive modeling for COVID-19 cases and deaths in the U.S. PLoS ONE. 2024;19:e0302324. 38843223 10.1371/journal.pone.0302324 PMC11156282

[2] Clinical Management. *of COVID-19: Living Guideline*World Health Organization, Geneva,. (2022). 35917394

[3] Peckham H, et al. Male sex identified by global COVID-19 meta-analysis as a risk factor for death and ITU admission. Nat Commun. 2020;11:6317. 33298944 10.1038/s41467-020-19741-6 PMC7726563

[4] Fabião J, et al. Why do men have worse COVID-19-related outcomes? A systematic review and meta-analysis with sex adjusted for age. Braz J Med Biol Res. 2022;55:e11711. 35195196 10.1590/1414-431X2021e11711 PMC8856598

[5] Takahashi T, et al. Sex differences in immune responses that underlie COVID-19 disease outcomes. Nature. 2020;588:315–20. 32846427 10.1038/s41586-020-2700-3 PMC7725931

[6] Jackson CB, Farzan M, Chen B, Choe H. Mechanisms of SARS-CoV-2 entry into cells. Nat Rev Mol Cell Biol. 2022;23:3–20. 34611326 10.1038/s41580-021-00418-x PMC8491763

[7] Deng Q, Rasool RU, Russell RM, Natesan R, Asangani IA. Targeting androgen regulation of TMPRSS2 and ACE2 as a therapeutic strategy to combat COVID-19. *iScience* 24, 102254 (2021). 10.1016/j.isci.2021.102254 PMC7919514 33681723

[8] Mikkonen L, Pihlajamaa P, Sahu B, Zhang F-P, Jänne OA. Androgen receptor and androgen-dependent gene expression in lung. Mol Cell Endocrinol. 2010;317:14–24. 20035825 10.1016/j.mce.2009.12.022

[9] Norman AW, Litwack G. Steroid hormones: Chemistry, Biosynthesis, and metabolism. In *hormones* (Academic).

[10] McCARTHY MM. Estradiol and the developing brain. Physiol Rev. 2008;88:91–134. 18195084 10.1152/physrev.00010.2007 PMC2754262

[11] Shabbir S, Hafeez A, Rafiq MA, Khan MJ. Estrogen shields women from COVID-19 complications by reducing ER stress. Med Hypotheses. 2020;143:110148. 32759016 10.1016/j.mehy.2020.110148 PMC7390780

[12] Washington NL, et al. Emergence and rapid transmission of SARS-CoV-2 B.1.1.7 in the United States. Cell. 2021;184:2587–e25947. 33861950 10.1016/j.cell.2021.03.052 PMC8009040

[13] Korber B, et al. Tracking changes in SARS-CoV-2 spike: evidence that D614G increases infectivity of the COVID-19 Virus. Cell. 2020;182:812–e82719. 32697968 10.1016/j.cell.2020.06.043 PMC7332439

[14] Bursac Z, Gauss CH, Williams DK, Hosmer DW. Purposeful selection of variables in logistic regression. Source Code Biol Med. 2008;3:17. 19087314 10.1186/1751-0473-3-17 PMC2633005

[15] DeLong ER, DeLong DM, Clarke-Pearson DL. Comparing the areas under two or more correlated receiver operating characteristic curves: a nonparametric approach. Biometrics. 1988;44:837–45. 3203132

[16] US Preventive Services Task Force. Hormone therapy for the primary Prevention of Chronic conditions in Postmenopausal persons: US Preventive Services Task Force Recommendation Statement. JAMA. 2022;328:1740. 36318127 10.1001/jama.2022.18625

[17] Matsuyama S et al. Enhanced isolation of SARS-CoV-2 by TMPRSS2-expressing cells. *Proc. Natl. Acad. Sci.* 117, 7001–7003 (2020). 10.1073/pnas.2002589117 PMC7132130 32165541

[18] Hoffmann M, et al. SARS-CoV-2 cell entry depends on ACE2 and TMPRSS2 and is blocked by a clinically proven protease inhibitor. Cell. 2020;181:271–e2808. 32142651 10.1016/j.cell.2020.02.052 PMC7102627

[19] Glowacka I, et al. Evidence that TMPRSS2 activates the severe Acute Respiratory Syndrome Coronavirus spike protein for membrane Fusion and reduces viral control by the Humoral Immune Response. J Virol. 2011;85:4122–34. 21325420 10.1128/JVI.02232-10 PMC3126222

[20] Mohamed MS, Moulin TC, Schiöth HB. Sex differences in COVID-19: the role of androgens in disease severity and progression. Endocrine. 2021;71:3–8. 33179220 10.1007/s12020-020-02536-6 PMC7657570

[21] Wambier CG, et al. Androgen sensitivity gateway to COVID -19 disease severity. Drug Dev Res. 2020;81:771–6. 32412125 10.1002/ddr.21688 PMC7273095

[22] Gebhard CE, et al. Sex versus gender-related characteristics: which predicts clinical outcomes of acute COVID-19? Intensive Care Med. 2022;48:1652–5. 35943570 10.1007/s00134-022-06836-5 PMC9361238

[23] Okwan-Duodu D, Lim E-C, You S, Engman DM. TMPRSS2 activity may mediate sex differences in COVID-19 severity. Signal Transduct Target Ther. 2021;6:100. 33649313 10.1038/s41392-021-00513-7 PMC7919249

[24] Bakker J, Baum MJ. Role for estradiol in female-typical brain and behavioral sexual differentiation. Front Neuroendocrinol. 2008;29:1–16. 17720235 10.1016/j.yfrne.2007.06.001 PMC2373265

[25] Stocco C. Tissue physiology and pathology of aromatase. Steroids. 2012;77:27–35. 22108547 10.1016/j.steroids.2011.10.013 PMC3286233

[26] Stanelle-Bertram S, et al. CYP19A1 mediates severe SARS-CoV-2 disease outcome in males. Cell Rep Med. 2023;4:101152. 37572667 10.1016/j.xcrm.2023.101152 PMC10518605

[27] Li F, et al. Estrogen hormone is an essential sex factor inhibiting inflammation and immune response in COVID-19. Sci Rep. 2022;12:9462. 35676404 10.1038/s41598-022-13585-4 PMC9175532

[28] Sund M, Fonseca-Rodríguez O, Josefsson A, Welen K. Fors Connolly, A.-M. Association between pharmaceutical modulation of oestrogen in postmenopausal women in Sweden and death due to COVID-19: a cohort study. BMJ Open. 2022;12:e053032. 35165090 10.1136/bmjopen-2021-053032 PMC8844968

[29] Lott N, et al. Sex hormones in SARS-CoV-2 susceptibility: key players or confounders? Nat Rev Endocrinol. 2023;19:217–31. 36494595 10.1038/s41574-022-00780-6 PMC9734735

[30] Salciccia S, et al. Interplay between male testosterone levels and the risk for subsequent invasive respiratory assistance among COVID-19 patients at hospital admission. Endocrine. 2020;70:206–10. 33030665 10.1007/s12020-020-02515-x PMC7543668

[31] Rastrelli G, et al. Low testosterone levels predict clinical adverse outcomes in SARS-CoV‐2 pneumonia patients. Andrology. 2021;9:88–98. 32436355 10.1111/andr.12821 PMC7280645

[32] Camici M, et al. Role of testosterone in SARS-CoV-2 infection: a key pathogenic factor and a biomarker for severe pneumonia. Int J Infect Dis. 2021;108:244–51. 34023492 10.1016/j.ijid.2021.05.042 PMC8135187

[33] Yassin A, et al. Testosterone and Covid-19: an update. Rev Med Virol. 2023;33:e2395. 36056748 10.1002/rmv.2395 PMC9537909

[34] Pugh PJ, Channer KS, Parry H, Downes T. Hugh Jones, T. BIO-AVAILABLE TESTOSTERONE LEVELS FALL ACUTELY FOLLOWING MYOCARDIAL INFARCTION IN MEN: ASSOCIATION WITH FIBRINOLYTIC FACTORS. Endocr Res. 2002;28:161–73. 12489566 10.1081/erc-120015055

[35] Manwani B, et al. Increased P450 aromatase levels in post-menopausal women after acute ischemic stroke. Biol Sex Differ. 2021;12:8. 33413673 10.1186/s13293-020-00357-w PMC7792154

[36] Bashar NAS, Gohar NMA-H, Tantawy AA, Kamel MH. M. evaluation of relationship between TMPRSS2 p.(Val197Met) variant and COVID-19 susceptibility and severity. BMC Infect Dis. 2024;24:112. 38254046 10.1186/s12879-024-08987-w PMC10802041

[37] Strope JD, PharmD CHC, Figg WD. TMPRSS2: potential biomarker for COVID-19 outcomes. J Clin Pharmacol. 2020;60:801–7. 32437018 10.1002/jcph.1641 PMC7280622

[38] Metzdorf K, et al. TMPRSS2 is essential for SARS-CoV-2 Beta and omicron infection. Viruses. 2023;15:271. 36851486 10.3390/v15020271 PMC9961888

